# Massive Ascites as the Only Sign of Ovarian Juvenile Granulosa Cell Tumor in an Adolescent: A Case Report and a Review of the Literature

**DOI:** 10.1155/2013/386725

**Published:** 2013-01-29

**Authors:** Azin Ashnagar, Samin Alavi, Yalda Nilipour, Roxana Azma, Farahnaz Falahati

**Affiliations:** ^1^Pediatric Congenital Hematologic Disorders Research Center, Shahid Beheshti Medical University, Tehran, Iran; ^2^Department of Pediatric Pathology, Mofid Children's Hospital, Shahid Beheshti Medical University, Iran; ^3^Department of Pediatric Radiology, Mofid Children's Hospital, Shahid Beheshti Medical University, Iran; ^4^Tehran University of Medical Sciences, Iran

## Abstract

Ovarian neoplasms are relatively rare in childhood and adolescence; only 5% to 8% of the cases are of sex cord stromal origin. Granulosa cell tumors are a group of estrogen producing sex cord stromal tumors of the ovary. They occur in 95% of the cases in adults, and only about 5% of the cases, which differ in histologic characteristics, are of juvenile type. A 13-year-old girl is reported who presented with massive abdominal distention and ascites. An abdominopelvic computed tomography scan showed a predominantly cystic mass lesion with septations arising from the left ovary. All tumor markers were normal, but serum inhibin level was increased. The patient underwent mass resection with salpingoophorectomy. Histopathology was compatible with the juvenile granulosa cell tumor. Interestingly, menarche was started in the patient soon after the surgery. To the best of our knowledge, massive ascites as the only clinical manifestation in the juvenile granulosa cell tumor has not reported as yet.

## 1. Introduction

Granulosa cell tumors (GCTs) are of sex cord stromal origin and account for approximately 2% of all ovarian tumors [1, 2]. They are predominantly made up of granulosa cells, theca cells, and fibroblasts in varying degrees and combinations. Based on histologic characteristics and age of onset, these tumors may be subdivided into adult type (95% of the cases) and juvenile type (5% of the cases) [3].

The juvenile granulosa cell tumor (JGCT) is an uncommon tumor that arises from nongerminative tissue of the ovary and typically occurs in the first two decades of life [4, 5]. This hormonally active ovarian neoplasm is an estrogen-producing tumor which may lead to its early detection. Common presentations associated with hormonal changes are precocious pseudopuberty, vaginal bleeding, irregular menstruation, and rarely virilization or hirsutism. Other common symptoms are abdominal pain and abdominal distention [6]. The mean age of diagnosis of JGCT is before the first decade of life, accounting for only one percent of all ovarian tumors. GCT is especially rare in children and only 4-5 percent of its cases occur in childhood [7]. JGCT is histopathologically and clinically distinct from the granulosa cell tumor which often occurs in premenopausal women. It has a favorable prognosis and is usually limited to one ovary [6, 8]. Herein, the occurrence of JGCT with an unusual presentation of massive ascites in a 13-year-old girl is reported.

## 2. Case Report

**Figure 1:**
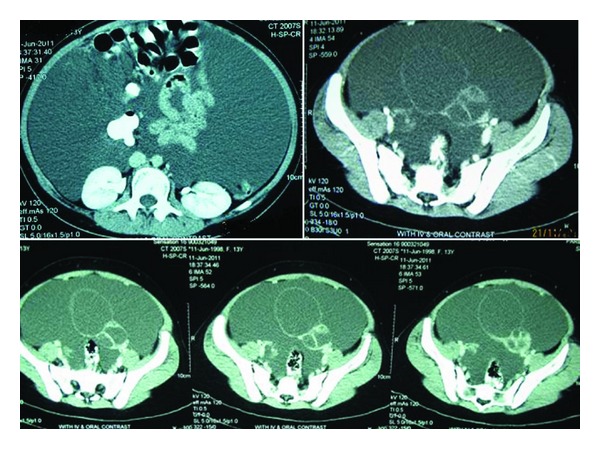
A 10 × 6 cm, multiseptated cystic mass is seen in the pelvis, originating from the left ovary with midline crossing associated with a large amount of ascites in the abdominopelvic cavity. No evidence of tumoral implants is seen on the peritoneal and mesenteric surfaces.

A 13-year-old girl presented with a progressive painless abdominal distention for almost two months. She had not experienced any menstruation cycles before. Physical examination revealed shifting dullness on a very distended abdomen. Abdominopelvic ultrasonography showed increased amounts of ascites and a cystic lesion with few septations in the pelvic cavity that measured 70 mm × 64 mm in greatest diameter and seemed to have mostly arisen from the left adnexa. Right ovary was also enlarged with a cystic lesion of 36 mm in maximum diameter. Massive amounts of ascites in the abdominopelvic cavity were present. Abdominopelvic CT scan demonstrated a cystic mass lesion arising from the left ovary with multilocular thick-walled components and a large cystic component extending to the right side of the pelvic cavity. There was also large amounts of ascites (Figure 1). The levels of *β*-hCG, AFP, CEA, and CA 19–9, CA-125 were measured preoperatively which were all within the normal range. Tumor resection with left salpingoophorectomy and bilateral omentectomy was performed through which about 10 liters of transudate ascites fluid was evacuated from the abdomen. Cytology of ascitic fluid was negative for malignancy. 

**Figure 2:**
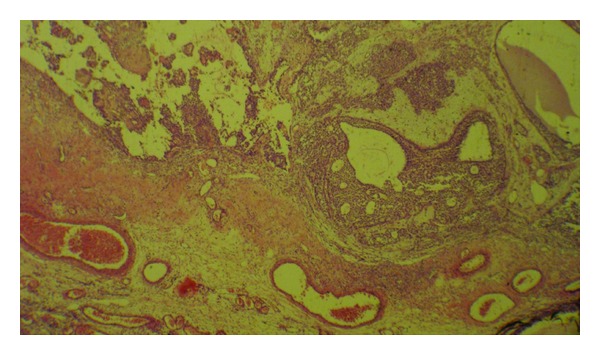
Solid and cystic pattern of the tumor with irregular follicles and pseudopapillary projections (H&E, ×100).

**Figure 3:**
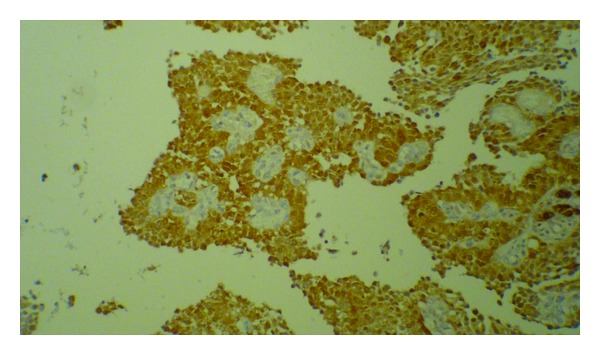
Immunohistochemical labeling of tumor cells with calretinin.

**Figure 4:**
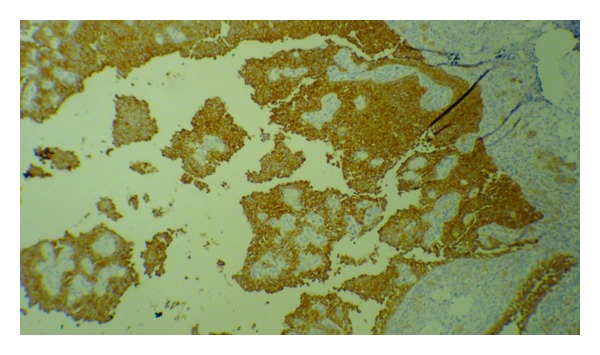
Immunohistochemical labeling of tumor cells with inhibin.

Grossly, there was a tumor on the surface of the ovary without any obvious capsule associated with the attached oviduct. Tumor was a large predominantly cystic multilocular lesion measuring 9 × 5 × 3.5 cm filled with clear yellow fluid and scattered papillary projections. Histopathology revealed ovarian tissue partially replaced by a neoplasm of complex growth pattern with nodules of ovoid and polygonal pale eosinophilic to clear cells as well as multiple irregular follicles with cystic changes in some of them and proliferations of granulosa-like cells in the ovarian stroma associated with pseudopapillary projections compatible with Juvenile granulosa cell tumor (Figure 2). Immunohistochemical analysis of tumor was positive for calretinin (Figure 3), inhibin (Figure 4), and CD99. Chest CT-scan and bone survey were unremarkable. Based on the FIGO staging system for ovarian tumors, the patient was classified as stage IC [9]. In our patient, serum inhibin level was not assessed before surgery, because the diagnosis of GCT was not suspected. However, there was an increased level of inhibin measured immediately after the surgery. Based on the results of the follow-up imaging postoperatively which showed evidence of residual tumor in the pelvic cavity and serum inhibin level, the patient was scheduled to receive 4 cycles of adjuvant chemotherapy with carboplatin, bleomycin, etoposide, and vinblastine. Follow-up imaging studies after commencing chemotherapy came as normal. The patient who had not experienced menstruation before developed a normal pattern of menarche shortly after the surgery. She was in complete remission without any residual tumor in an 18-month follow-up period. 

## 3. Discussion

Sex cord stromal tumors comprise about 5–8% of all malignant ovarian neoplasms. The incidence of GCTs which is a subset of sex cord stromal tumors varies from 0.4 to 1.7 cases per 100,000 women [10, 11]. Only about 5% of GCT cases which differ in histologic characteristics are of juvenile type [4]. Most of JGCTs occur in prepubertal girls [12, 13]. In a study on 125 cases of JGCT, 44% of the tumors occurred before the first decade of life and only 3% developed after the third decade [13]. The majority of prepubertal patients who are afflicted with secretary GCTs present with clinical evidence of precocious pseudopuberty including breast enlargement, development of pubic and axillary hair, vaginal secretions, irregular uterine bleeding, somatoskeletal changes, and other secondary sex characteristics [12–15]. However, our case was free of such symptoms and her presentation was mainly massive ascites leading to the sudden enlargement of the abdomen. Furthermore, she started to have menstruation some 10 days after the operation whereas she had not achieved the menarche before. There are two reported cases of primary amenorrhea who started menstruation shortly after resection of GCT [4, 7]; however, JGCT presenting with massive ascites similar to this patient has not been addressed anywhere. Increased serum inhibin and subsequent FSH suppression have been proposed as causative agents for primary amenorrhea [4]. 

The natural history of GCT is indolent with a very favorable long-term prognosis similar to that of mildly malignant tumors. The optimal management of sex cord ovarian tumors is limited by their low incidence, the multiplicity of their histologic patterns, and their variable biologic behavior [14]. 

As for tumor markers, the measurement of serum AFP and *β*-hCG level is important to exclude secreting germ cell tumors [14], which were normal in the presented case. Inhibin is a peptide hormone produced by ovarian granulosa cells that has a regulatory effect on FSH secretion. Patients with both adult and juvenile types of GCTs have been shown to have increased levels of inhibin which may return to the baseline levels postoperatively [16]. 

Among all clinical parameters, tumor stage is of greatest prognostic value and must be carefully assigned for all patients. Patients with JGCT typically present at an early stage and enjoy a favorable prognosis; however, those with more advanced-stage disease (FIGO stages II, III, or IV) may experience an aggressive clinical course with a short remission to relapse or death [10, 14]. Both juvenile- and adult-type GCTs are almost always unilateral [12–14]. According to the unilateral origin of the tumor, tumor projections on the surface of the ovary, and negative cytology of the ascitic fluid, our case was considered as stage 1C [9]. 

The mainstay of treatment of JGCT is surgery which is often modified to unilateral salpingoophorectomy to preserve fertility in the child-bearing women once extra-ovarian spread has carefully been ruled out [10, 14]. These tumors are often not suspected until frozen sections are made, and since frozen sections are fraught with many inaccuracies, their diagnosis requires a great deal of expertise [14]. Despite being less differentiated than the adult form, JGCTs tend to have a high cure rate as indicated by the follow-up data [10]. 

Prospective randomized studies have so far failed to establish the benefits of the postoperative adjuvant therapy for high-risk patients. Patients with advanced stages or recurrent disease are currently treated with platinum-based chemotherapy and reports on their effectiveness suggest an overall response rate of 63% to 80% [17]. However, some trials on taxane and platinum combination chemotherapy are also underway. No sound body of evidence on the possible benefits of radiation or hormonal therapy is currently available, and therefore their use may only be justified in special cases [18]. In our patient according to the residual tumor observed on postoperative imaging and persistent large amounts of ascites fluid, chemotherapy with 4 courses of ICE began for the patient. 

Late relapse of GCT mandates prolonged followups. The 5-year survival rate for patients with stage I GCT as reported by different authors is above 90%; however, the figure has also been reported to be as low as 75% [19]. In addition to the rarity of ovarian GCTs in childhood, the presented case is of particular interest for atypically having abdominal distention due to massive ascites as the sole presenting symptom. It may also be a reminder of our need to further investigate different aspects of this tumor.
